# The effect of a 12-week resistance training intervention on leukocyte telomere length

**DOI:** 10.1016/j.heliyon.2020.e04151

**Published:** 2020-06-10

**Authors:** Matt Nickels, Sarabjit Mastana, David Hunter, Matthew Denniff, Veryan Codd, Elizabeth Akam

**Affiliations:** a School of Sport, Exercise and Health Sciences, Loughborough University, Loughborough, Leicestershire, LE11 3TU, United Kingdom; b Department of Cardiovascular Sciences, University of Leicester, Leicester, United Kingdom; c NIHR Leicester Cardiovascular Biomedical Research Unit, Glenfield Hospital, Leicester, LE3 9QP, United Kingdom

**Keywords:** Molecular dynamics, Aging, Physical activity, Human genetics, Physiology, Exercise, Tumour necrosis factor alpha, Strength, BODYPUMP

## Abstract

Telomere dynamics are an active biological process and positive lifestyle factors such as exercise are proposed to potentiate their length. The aim of this study was to investigate the effect of a low-resistance, high-repetition resistance training intervention on leukocyte telomere length (LTL) and associated health parameters. 23 sedentary middle-aged adults volunteered for this study (16 female/7 male; age = 51.5 ± 4.9 years) and performed two one-hour sessions of Les Mills BODYPUMP™ per week for 12 weeks. Outcome measures were taken at baseline, after the training intervention and at 12-month follow-up. LTL remained unchanged following the training intervention (pre 0.819 ± 0.121 vs post 0.812 ± 0.114, p = 0.420), despite a borderline significant increase in hTERT expression (p = 0.050). Circulating levels of tumour necrosis factor alpha were reduced after the intervention (p = 0.001). At 12-month follow-up, subjects who returned to a sedentary lifestyle (n = 10) displayed shorter telomeres compared to their pre (p = 0.036) values. In conclusion, no changes were observed in LTL following the 12-week training intervention, despite improvements in molecular parameters associated with telomere dynamics. It appears continued long-term exercise (>12 months) is necessary to preserve LTL in previously sedentary individuals.

## 1 Introduction

Telomeres are repeat, non-coding DNA sequences located at chromosome ends. They function as protective caps that confer genomic stability, protecting internal regions from deterioration, end-to-end fusion and recombination (Neidle and Parkinson, 2003). Telomere length (TL) declines with age and is considered a key biomarker of biological aging. Short telomeres are associated with numerous age-related diseases, such as cardiovascular disease, diabetes and several types of cancer (Rizvi et al., 2015). When a cells telomeres become critically short it ceases to divide and undergoes replicative senescence (Harley et al., 1990).

Inflammation and oxidative stress are two primary factors identified to accelerate telomere attrition (Correia-Melo et al., 2014). Psychosocial, behavioural, and environmental factors that promote inflammation and/or oxidative stress are associated with shorter telomeres, such as obesity, smoking and psychological stress (Starkweather, 2014). Conversely, lifestyle behaviours which reduce inflammation and oxidative stress are positively associated with TL, such as habitual physical activity. Most studies to date have investigated the effects of aerobic endurance exercise on TL, primarily reporting a positive association (Borghini et al., 2015; Denham et al., 2013; LaRocca et al., 2010). Several cross-sectional studies have also shown a positive relationship between self-reported physical activity and TL (Bendix et al., 2011; Cherkas et al., 2008; Latifovic et al., 2016). However, research is currently lacking regarding the effects of different exercise modalities on TL, particularly resistance training.

Long-term resistance training ameliorates systemic inflammation and age-related decline in muscle strength (Calle and Fernandez, 2010; Mayer et al., 2011). Furthermore, greater lower extremity muscle strength has been linked with longer leukocyte telomere length (LTL) (Dimauro et al., 2016; Loprinzi and Loenneke, 2016), highlighting the potential of resistance training to influence telomere dynamics. In support of the possible association between resistance training and TL, 12 weeks of low frequency, moderate intensity, explosive-type resistance training prevented telomere shortening (Dimauro et al., 2016). However, TL was unchanged in both sedentary and elderly individuals following separate 6-month resistance training interventions (Tosevska et al., 2016; Werner et al., 2018).

The aim of the present study was to investigate the effects of a 12-week low-resistance, high-repetition resistance training on LTL and inflammatory markers in sedentary middle-aged individuals.

## 2 Materials and methods

### 2.1 Ethical approval

Ethical approval for this study was provided by the Loughborough University ethical advisory committee (R18_P075). Written informed consent was obtained from all participants and the study conformed to the standards detailed by the latest revision of the Declaration of Helsinki.

### 2.2 Participants

Twenty-three untrained healthy subjects voluntarily participated in the present study (16 female/7 male: age = 51.5 ± 4.9 years, height = 166.3 ± 9.3 cm, weight = 78.4 ± 14.7 kg, BMI = 28.0 ± 4.6 kg/m^2^, body fat = 34.6 ± 9.2%). The health status, physical activity level (International Physical Activity Questionnaire - Short Form (IPAQ-SF)) and mental wellbeing (The Warwick-Edinburgh Mental Wellbeing Scale (WEMWBS)) of each subject were assessed by questionnaires prior to testing. Inclusion criteria for the study were no known metabolic disorders, cardiovascular disease or cancer, non-smokers, no use of anti-inflammatory medication or steroids. All subjects were considered sedentary prior to the study (based on a low score on the IPAQ-SF (<600 MET minutes/week)) and had no or minimal previous resistance training experience. Following the resistance training intervention, subjects were considered recreationally active if they scored moderate (600–3000 MET minutes/week) or high (>3000 MET minutes/week) on the IPAQ-SF. Subjects who scored low on the IPAQ-SF were considered to have returned to a sedentary lifestyle. An additional question required subjects to confirm that they had maintained their calculated IPAQ-SF score for at least 11 months of the subsequent 12-month period in order to accurately reflect their physical activity level for the previous year.

### 2.3 Experimental design and training protocol

All subjects were required to attend a laboratory testing session the week before (pre) and a week after (post) the 12-week resistance training intervention. The order of the tests was the same for all subjects and both testing sessions were performed at the same time of the day to minimise any impact of diurnal variation. The testing protocol for all subjects was as follows: anthropometric measurements, blood sample collection, physical performance measures (countermovement jump, isometric leg strength, grip strength, chair stand test).

Subjects attended two 1-hour sessions of Les Mills BODYPUMP™ per week for 12 weeks. Sessions were held at the same time of day and conducted on two non-consecutive days by a Les Mills licensed instructor. Les Mills BODYPUMP™ is a resistance training class demonstrated to improve muscular strength and decrease metabolic stress, but has no influence on running aerobic fitness (Greco et al., 2011). Training sessions involved subjects using a self-selected light to moderate weight as part of a low-resistance, high-repetition total body workout that was continually adjusted according to the perception of the subjects and instructor. Each session was performed using the pre-defined BODYPUMP™ exercise programme which consists of 10 exercise-music selections, each lasting 4–6 min, for approximately 60 min. Detailed information regarding exercise order and duration is presented elsewhere (Greco et al., 2011).

Subjects were recalled for anthropometric measurements, questionnaires and blood sample collection a year after their post resistance training intervention tests as part of a 12-month follow-up (12-MFU) visit.

### 2.4 Anthropometric measurements

Height and weight were measured using a portable Seca 213 stadiometer (Seca GmbH, Germany) and calibrated Adam CFW-150 digital scale (Adam Equipment Ltd, UK), respectively. Estimates of body fat percentage (BF%), fat mass (FM), fat-free mass (FFM), total body water (TBW), body mass index (BMI) and visceral fat rating were quantified using the Tanita BC-418 MA segmental body composition analyser (Tanita Corporation, USA).

### 2.5 Physical performance parameters

#### 2.5.1 Warm up

All subjects performed a standardised warm up prior to physical testing; stepping up and down on a 30cm high step, one foot at a time, to a metronome beat at 15 steps/minute for 3 min. The step rate then increased to 20 steps/minute for another 3 min, for a total time of 6 min.

#### 2.5.2 Countermovement jump

All tests were conducted on a JumpMat Pro contact mat (JumpMat, Australia). Subjects were given verbal instructions and a visual demonstration of the countermovement jump (CMJ) prior to testing and were allowed two practice jumps. Standing on the contact mat, subjects were instructed to jump as high as possible and attempt to land in the same position as they took off. The countermovement drop depth was standardised to parallel for all trials, unless the subject could not reach depth. Subjects were required to keep their hands on the hips throughout the test and maintain extension in the hip, knee, and ankle joints during flight-time. Each subject performed three recorded test jumps and the greatest jump height, recorded to the nearest 0.1cm, was used in subsequent analysis.

#### 2.5.3 Grip strength

Isometric strength of the hand and forearm muscles was measured using a Takei hand grip dynamometer (Takei Equipment Industry Company, Japan). The dynamometer was adjusted so that it was comfortable in the subject's hand and the handle position was recorded to ensure consistency between pre- and post-intervention measurements. Testing was conducted with participants seated, elbow by their side and flexed to 90^o^, forearm in 0° between pronation and supination and a neutral wrist position. Testing began with the dominant hand and subjects squeezed the dynamometer with maximum isometric effort for 3–5 s with verbal encouragement. Each subject performed 3 tests per hand in alternative fashion, resting 30 s between attempts. The maximum value for each hand was recorded in kilograms and used for data analysis.

#### 2.5.4 Isometric leg strength

Isometric leg strength was measured using a Takei back and leg dynamometer (Takei Equipment Industry Company, Japan). Following a visual demonstration subjects stood on the footplate of the dynamometer with their knees flexed until the leg extension angle measured 120^o^. With their elbows fully extended, subjects were instructed to pull the bar in a vertical direction with maximal effort while maintaining the stable initial starting position (no 'jerking' movements or breaking at the knees). The maximal score from three attempts was used for subsequent data analysis.

#### 2.5.5 Chair stand test

The chair stand test required subjects to stand up from and sit down on an armless chair as quickly as possible 10 times. Subjects folded their arms across their chests and were instructed to stand-up completely and make firm contact with their lower back when sitting. A three-second countdown was given to the subjects, with timing commencing on the word ‘start’ and ceasing when the subject sat after the tenth repetition. Subjects were permitted 2 practice repetitions before the single test trial.

### 2.6 Sample collection and processing

A trained phlebotomist collected blood the week before and after the 12-week resistance training intervention. Additional blood samples were taken 12 months after a participant post visit. All bloods were taken at least 24 h post-exercise. Whole blood for DNA extraction was collected in a dipotassium ethylenediaminetetraacetic acid (K2EDTA) vacutainer (Becton, Dickson & Company, UK) and aliquoted directly into Eppendorf's to be stored at −80 °C. Differential cell count analysis was performed with EDTA anticoagulated blood within 30 min of withdrawal using a Yumizen H500 (Horiba Medical, Japan). Anticoagulant-free vacutainers (Becton, Dickson & Company, UK) were used for serum isolation and kept at room temperature for 30–60 min prior to centrifugation at 1400xg (4100 rpm) for 5 min (20 °C). The collected material was recentrifuged using the same conditions to ensure the complete removal of any residual cells. Serum was removed and aliquots were stored at −80 °C. For RNA analysis, whole blood was collected in Tempus tubes (Applied Biosystems, USA) which were inverted several times to mix the collected blood. Tubes were then kept at room temperature for approximately 15 min before transfer to −80 °C, where they were stored until analysis.

### 2.7 DNA extraction and telomere length analysis

DNA was extracted from whole blood using the commercial ReliaPrep™ Blood gDNA Miniprep System (Promega, USA) according to the manufacturer's instructions. The quantity and quality of genomic DNA isolate was assessed by spectrophotometry using a NanoDrop 2000 (ThermoFisher, USA), producing acceptable A260/280 values (1.93 ± 0.02). TL was measured in leukocytes and conducted at the University of Leicester using a modified protocol based on the qPCR method previously described (Cawthon, 2002). Telomere primer sequences were Tel1b 5′-CGGTTTGTTTGGGTTTGGGTTTGGGTTTGGGTTTGGGTT-3’; Tel2b 5′-GGCTTGCCTTACCCTTACCCTTACCCTTACCCTTACCCT-3′, both diluted to a final concentration of 300nM. Primer sequences for the single-copy gene (36B4) were 36B4F 5′-CAGCAAGTGGGAAGGTGTAATCC -3′; 36B4R 5′-CCCATTCTATCATCAACGGGTACAA -3′, both diluted to a final concentration of 500nM. Final reaction volume was 25μL, consisting of 22μL of qPCR master mix (2x SensiMix NoRef DNA kit, Bioline) and primers (targeting either the telomere or single copy gene) and 3μL of gDNA template (10 ng/μL). Telomere (T) PCR cycling conditions consisted of 95 °C for 10 min, 20 cycles of 95 °C for 15 s and 58 °C for 1 min. Single-copy gene (S) PCR cycling conditions consisted of 95 °C for 10 min, 30 cycles of 95 °C for 15 s and 58 °C for 1 min. Telomere and single copy gene runs were performed separately, starting with former and followed immediately by the latter to keep conditions uniform. To ensure intra-run reliability each sample was run in duplicate. Any duplicate samples which had take-off values exceeding 0.2 of a cycle difference were excluded and re-run. To ensure inter-run reliability all PCR reactions were performed twice on two separate days using a Rotorgene Q (Qiagen, Germany). The average inter-plate coefficient of variation for calculated T/S ratio was 5.1%. Relative T/S ratios were calculated using the Qiagen Rotorgene comparative quantitation software (Qiagen, Germany).

### 2.8 RNA extraction and analyses of gene expression by quantitative PCR

RNA was extracted from whole blood collected in Tempus Blood RNA tubes using the commercial Tempus™ Spin RNA Isolation Kit (Applied Biosystems, USA) according to the manufacturer's instructions. The quantity and quality of genomic RNA isolate was assessed by spectrophotometry using a NanoDrop 2000 (ThermoFisher, USA), producing acceptable A260/280 values (2.12 ± 0.01). A minimum of 800ng RNA was reverse-transcribed to cDNA using the High-Capacity Reverse Transcription kit (Applied Biosystems, USA) according to the manufacturer's instructions. cDNA was diluted to a final concentration of 5 ng/μL using RNAse/DNAse free water. Relative mRNA expression level of human telomerase reverse transcriptase (hTERT) was normalised to the geometric mean of housekeeper genes Cytochrome C1 (CYC1), ATP synthase F1 subunit beta (ATP5B) and Glyceraldehyde 3-phosphate dehydrogenase (GAPDH) using a Viia7 Real-Time PCR system (Applied Biosystems, USA). Each reaction contained 5μL of SybrGreen PrecisionPlus qPCR Master Mix (PrimerDesign, UK), 0.5μL of primer mix and 4μL of 5 ng/μL cDNA, with the total volume made up to 10μL using RNAse/DNAse free water. All samples were run in duplicate using the following cycling conditions: denaturation at 95 °C for 2 min, followed by 40 cycles of 95 °C for 10 s and 60 °C for 60s. Melt curves were visually inspected for a single peak indicating the generation of a single product. The relative mRNA expression for hTERT was calculated using the 2^−(ΔΔCt)^ formula; the pooled mean pre-intervention Ct from all participants was used as the control. Values 2^−(ΔΔCt)^ were log transformed prior to statistical analysis to avoid any abnormal distribution. The mean Ct values of GAPDH, ATP5B and CYC1 across all participants and experimental conditions were 14.84 ± 0.88, 18.54 ± 0.53 and 22.18 ± 0.44, respectively.

### 2.9 Measurement of inflammatory markers

Inflammatory markers interleukin 6 (IL-6), interleukin 8 (IL-8), tumour necrosis factor alpha (TNF-α) and C-reactive protein (CRP) were all measured in serum. IL-6, IL-8 and TNF-α levels were measured in duplicate by enzyme-linked immunosorbent assay (ELISA) kits (R&D Systems, USA) according to the manufacturer's instructions. The average intra-assay coefficient of variation (CV) was 5.3 ± 1.5% for the different assays. The average inter-assay CV was 3.4 ± 2.1% for the different assays. Serum levels of CRP were measured on a single run using a commercially available spectrophotometric assay (Horiba Medical, UK) and a Pentra 400 semiautomated analyser (Horiba Medical, UK). The average intra-assay CV for CRP measurement was 1.5 ± 0.1%.

### 2.10 Statistical analysis

Wilcoxon signed-rank test was used to compare pre- and post-intervention data (e.g. TL, hTERT expression, inflammatory markers, physical performance measures and body composition), circumventing the requirement of making assumptions about the distributions of variables in a small sample size. Spearman's test was used to verify correlations. Differences within groups at 12-MFU were determined using a repeated measures ANOVA and a Bonferroni correction. If data violated the assumption of sphericity, a repeated measures ANOVA with a Greenhouse-Geisser correction was used. All analyses were performed using SPSS 23.0 (IBM Corporation, USA) with p-values < 0.05 considered significant.

## 3 Results

### 3.1 Telomere length

**Figure 1:**
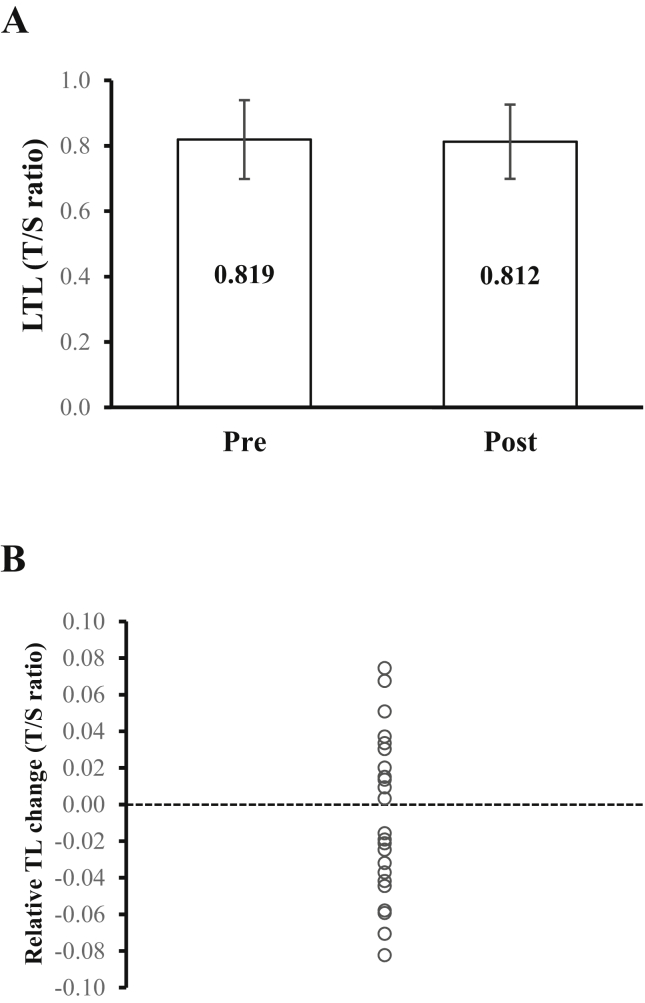
(A) Pre and post leukocyte telomere length (LTL) (mean ± SD) and (B) inter-individual relative TL change following the 12-week resistance training intervention. Data are presented as T/S ratio.

A Wilcoxon signed-rank test showed that there was no significant difference between mean pre- and post-intervention LTL values (Z = -.806, p = 0.420) (Figure 1a). Inter-individual responses revealed telomere shortening in 52% of subjects and lengthening in 48% following the resistance training intervention (Figure 1b). Spearman's rank-order correlation revealed age was not associated with LTL at pre (*r*_*s*_ = -.135, p = .539) or post (*r*_*s*_ = -.210, p = .337) timepoints. Therefore, no further adjustments for age were carried out.

### 3.2 Inflammatory markers

Subjects displayed significantly reduced levels of serum TNF-α following the 12-week intervention. No other inflammatory markers showed any significant change (Table 1).

**Table 1:** Levels of inflammatory markers at baseline (pre) and following (post)12-weeks low-resistance high-repetition resistance training.

	pre	post	Δ post vs. pre	p
TNF-α (pg/ml)	0.98 ± 0.41	0.80 ± 0.37	-0.18 ± 0.25	**0.001∗**
IL-6 (ng/ml)	3.20 ± 0.36	3.52 ± 0.56	0.32 ± 1.73	0.761
IL-8 (ng/l)	10.92 ± 0.79	10.28 ± 0.59	-0.64 ± 3.09	0.211
CRP (mg/l)	3.06 ± 1.01	2.77 ± 0.93	-0.29 ± 1.56	0.637

Data are means ± SD. ∗Statistical significance defined as p-values <0.05, Wilcoxon signed-rank test and expressed to three decimal places.

Significant inverse associations were found between CRP level and LTL at pre and post timepoints. LTL was not associated with any other inflammatory marker (Table 2).

**Table 2:** Spearman correlation coefficients regarding inflammatory markers and telomere length observed before (pre) and after (post) the 12-week resistance training intervention.

	pre		post	
	r_s_	p	r_s_	p
TNF-α (pg/ml)	-.309	.151	-.269	.214
IL-6 (ng/ml)	-.277	.200	-.025	.911
IL-8 (ng/l)	-.105	.634	-.051	.816
CRP (mg/l)	-.437	**.037∗**	-.439	**.036∗**

r_s_, Spearman correlation coefficient. ∗Statistical significance defined as p-values <0.05.

### 3.3 hTERT mRNA expression level

**Figure 2:**
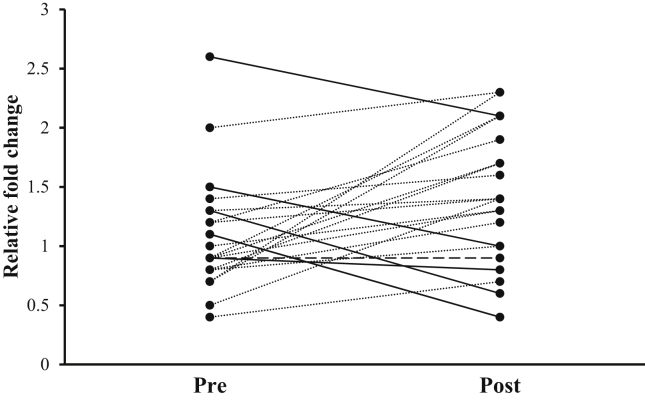
Inter-individual responses of hTERT mRNA relative fold change following a 12-week resistance training intervention. Gene expression data is expressed relative to the geometric mean of housekeeper genes CYC1, ATP5B and GAPDH. Solid line indicates decrease (n = 5), dotted line indicates increase (n = 16) and dashed line indicates no change (n = 1). Data was lost for one subject during sample preparation.

There was no change in the mean relative mRNA expression level of hTERT following the resistance training intervention (average pre 2^−(ΔΔCt)^ values = 1.1 ± 0.5 vs average post 2^−(ΔΔCt)^ values = 1.4 ± 0.6; p = 0.050), although it was bordering a significant increase (Figure 2).

### 3.4 Body composition

There was a significant decrease in subject weight following the 12-week resistance training intervention. However, all other measures of body composition remained unchanged (Table 3). No measures of body composition were associated with LTL at any timepoint (p > 0.05), so no further adjustments were required.

**Table 3:** Body composition and physical performance measures at baseline (pre) and after 12 weeks resistance training (post).

	pre	post	Δ post vs. pre	p
**Body composition**				
Weight (kg)	78.4 ± 14.7	77.0 ± 14.6	-1.4 ± 1.6	**0.002**∗
BMI (kg/m^2^)	28.0 ± 4.6	27.9 ± 4.6	-0.1 ± 0.6	0.158
Body fat (%)	34.6 ± 9.2	33.9 ± 9.8	-0.7 ± 1.7	0.079
FM (kg)	27.3 ± 10.5	26.6 ± 10.9	-0.7 ± 1.7	0.070
FFM (kg)	50.3 ± 10.3	50.4 ± 10.2	0.1 ± 1.1	0.807
TBW (kg)	36.8 ± 7.5	36.9 ± 7.5	0.1 ± 0.8	0.794
Visceral fat rating	9	9	0 ± 0	0.564
**Physical performance**				
CMJ (cm)	18.6 ± 6.9	22.9 ± 7.8	4.3 ± 4.9	**0.001**∗
Grip strength (kg) (right)	28.9 ± 9.9	31.6 ± 8.7	2.7 ± 4.2	**0.012**∗
Grip strength (kg) (left)	27.3 ± 9.8	29.2 ± 7.7	1.9 ± 4.5	0.097
Isometric leg strength (kg)	83.3 ± 38.5	93.4 ± 36.0	10.1 ± 16.5	**0.009**∗
Sit-to-stand test (s)	24 ± 6	19 ± 4	5 ± 5	**<0.001**∗

Data are means ± SD; BMI, body mass index. FM, fat mass. FFM, fat free mass. TBW, total body water. CMJ, countermovement jump. ∗Statistical significance defined as p-values <0.05, Wilcoxon signed-rank test, and expressed to three decimal places.

### 3.5 Physical performance parameters

There was a significant increase in mean physical performance scores for CMJ, grip strength (right), isometric leg strength and chair stand test following the resistance training intervention (Table 3). No physical performance measures were associated with LTL at any timepoint (p > 0.05).

### 3.6 Assessment of mental wellbeing

Subjects mental wellbeing significantly improved following the 12-week resistance training intervention (pre 47.7 ± 5.0 vs post 52.0 ± 6.0, p = 0.014), but was not associated with TL at any timepoint and required no further adjustments (p > 0.05).

### 3.7 12-month follow-up visit

**Figure 3:**
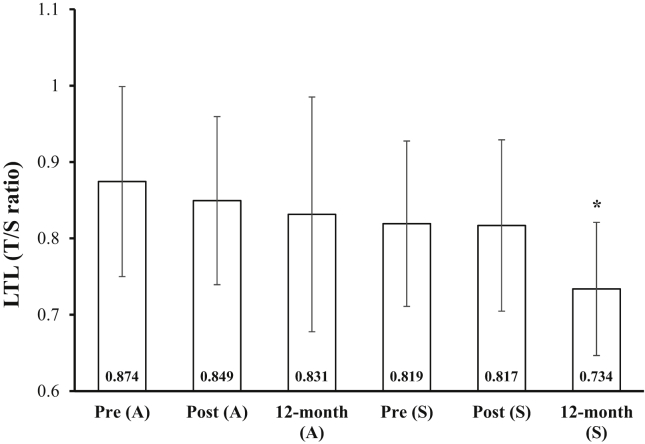
Pre, post and 12-month leukocyte telomere length (LTL) for active (A) and sedentary (S) groups (mean ± SD). Data are presented as T/S ratio. **∗**: different from Pre (S).

Individuals who returned to a sedentary lifestyle displayed significantly shorter telomeres at 12-MFU compared to their own pre intervention measurements (-0.85 T/S ratio, p = 0.036), but not their post values (-0.83 T/S ratio, p = 0.050), although this was approaching significance (Figure 3). A repeated measures ANOVA with a Greenhouse-Geisser correction determined that there was no difference in CRP (pre 3.02 ± 5.81, post 3.29 ± 5.63, 12-MFU 2.20 ± 2.93) or TNF-α (pre 0.844 ± 0.353, post 0.746 ± 0.292, 12-MFU 0.871 ± 0.284) at any time point in sedentary individuals (p > 0.05) (all measurements are presented as means ± SD). Circulatory CRP levels were not associated with LTL at any time point (p > 0.05). However, LTL was negatively associated with pre (r_s_ = -.758, p = 0.011) and post (r_s_ = -.748, p = 0.013) circulatory TNF-α levels in individuals who returned to a sedentary lifestyle, but not 12-MFU (p > 0.05).

Individuals who continued to exercise following the 12-week resistance training intervention showed no difference in TL (Figure 3), CRP (pre 0.81 ± 0.50, post 0.59 ± 0.31, 12-MFU 0.52 ± 0.22) or TNF-α (pre 1.21 ± 0.40, post 0.84 ± 0.19, 12-MFU 0.91 ± 0.09) levels between pre, post or 12-MFU (p > 0.05). Furthermore, LTL did not correlate with CRP at any time point (p > 0.05) but was surprisingly positively associated with TNF-α at 12-MFU (r_s_ = 0.900, p = 0.037). Pre and post levels TNF-α were not associated with LTL in individuals who remained active (p > 0.05).

## 4 Discussion

From pre and post analysis of a 12-week low-resistance, high-repetition resistance training intervention in healthy sedentary middle-aged subjects we provide evidence that: 1) no changes in TL were observed; 2) circulating TNF-α levels significantly decreased; 3) circulating CRP levels were inversely associated with TL. In addition, a 12-MFU showed that long-term (>12 months) regular exercise may act to preserve TL in previously sedentary middle-aged individuals. However, the present study only comprised a small sample size and the data should be interpreted with caution.

The results of the present study lend support to the null hypothesis that no observable changes in TL occurred following the 12-week resistance training intervention. This study is the first to longitudinally examine the effect of low-resistance, high-repetition resistance training on TL. There is a paucity of intervention studies investigating the relationship between TL and physical activity, with most of the research employing a cross-sectional design. Moreover, the current literature principally reports the effects of endurance aerobic exercise on TL. Among the few longitudinal studies that have assessed the influence of a resistance training intervention on telomere dynamics the results are inconclusive, with reported relationships of TL maintenance (Dimauro et al., 2016), no effect (Hagstrom, 2018; Tosevska et al., 2016; Werner et al., 2018) and TL shortening (Miranda-Furtado et al., 2015). The discrepant findings may be a consequence of variations in the frequency, volume, intensity and duration of the individual study resistance training protocols. It may be that ‘explosive-type’ resistance training, employed by Dimuaro et al., is a more influential form of resistance training regarding LTL maintenance (Dimauro et al., 2016). However, low-resistance, high-repetition resistance training did not appear to have any effect on LTL over a 12-week intervention period. Thus, our results support studies that report short-term resistance training interventions do not cause any observable changes in TL (Hagstrom, 2018; Tosevska et al., 2016; Werner et al., 2018).

Although it appears that the low-resistance, high-repetition resistance training employed in the present study does not influence LTL, it would be unreasonable to completely dismiss the potential benefits of this modality of exercise on telomere dynamics. Firstly, subjects who continued to exercise following the resistance training intervention did not display significant LTL loss at their 12-MFU visit, whereas subjects who reverted to a sedentary lifestyle showed significant telomere attrition from their pre-resistance training intervention values. However, the absence of significant telomere attrition in the exercise group cannot be solely attributed to resistance training, as subjects who continued to perform physical activity reported utilising various modalities in addition to resistance training (e.g. cycling, running, tennis). Nonetheless, inflammatory marker levels can be reduced by long-term resistance training (Calle and Fernandez, 2010), which is one of the main factors proposed to influence telomere attrition (Correia-Melo et al., 2014). Present results report that circulating levels of TNF-α were significantly reduced following the 12-week resistance training intervention. This finding is in line with data from Hagstrom et al. who found a significant reduction in natural killer and killer T-cell expression of TNF-α following a 16-week resistance training intervention (Hagstrom et al., 2016). Higher plasma concentrations of TNF-α have been previously reported to be inversely associated with TL (O'Donovan et al., 2011), a finding replicated in the sedentary subjects of the present study at 12-MFU. Thus, a decline in circulating TNF-α concentration suggests a potential mechanism by which low-resistance, high-repetition resistance training could positively influence telomere dynamics. A longer intervention period may have therefore been required in order to reveal a significant effect of resistance training on telomere dynamics.

The pro-inflammatory cytokine TNF-α usually acts as part of an inflammatory response. Initially secreted by a limited cell population, consisting of macrophages and T-cells, TNF-α exerts its effects via cognate receptors (TNF-RI and TNF-RII) which display a much wider cellular expression. This ubiquitous expression, in conjunction with cell-specific effector molecules that are triggered by receptor binding, may explain the variety of effects of TNF-α which include apoptosis, the synthesis of protein and lipid inflammatory molecules, and transcription factors. TNF-α-mediated inflammation contributes to chronic low-grade inflammation associated with advanced age (Franceschi et al., 2006) and is connected with many age-related diseases such as cardiovascular disease and rheumatoid arthritis (Rea et al., 2018). Linked to its pivotal role in the generation of inflammation, anti-TNF-α drug therapy has been successful in ameliorating several inflammatory conditions (Lopetuso et al., 2017). However, primary non-response, or loss of response, and negative side effects arise in certain individuals (Lopetuso et al., 2017). Thus, reducing TNF-α via low-resistance, high-repetition resistance training could be a viable method to delay inflammaging and associated diseases without the negative side effects of anti-TNF-α drugs.

Higher plasma concentrations of CRP have also been previously linked with shorter telomeres (O'Donovan et al., 2011; Rode et al., 2014), which our findings support. Furthermore, TNF-α and CRP levels are inversely associated with muscle mass (Visser et al., 2002) and strength (Westbury et al., 2018). To highlight a potential interconnected relationship between the factors, TL has been positively associated with measures of physical function, such as faster walking speed (Dimauro et al., 2016), CMJ height (Dimauro et al., 2016) and lower body muscle strength (Dimauro et al., 2016; Loprinzi and Loenneke, 2016), although the current literature is inconsistent regarding these relationships (Baylis et al., 2015; Mather et al., 2010). In the present study, only circulating TNF-α and CRP levels were inversely associated with LTL at various timepoints. Thus, our data agrees with previous studies that report physical function is not associated with TL, despite increases in performance measures following the resistance training intervention. Nevertheless, since long-term resistance training significantly reduces TNF-α and CRP levels (Santiago et al., 2018; Sardeli et al., 2018), this may provide a mechanistic explanation as to how resistance training delays age-related declines in LTL, muscle mass and strength and improves functional performance.

TL has been associated with multiple behavioural and psychosocial factors including diet, body composition, psychological stress and sleep (Starkweather, 2014). Thus, it is problematic isolating the effects of physical activity without controlling for these variables. In the present study we only measured body composition and mental wellbeing, neither of which were associated with LTL. Nevertheless, we cannot be sure that discrepancies in subjects’ diet and sleep patterns may have influenced our results. In addition to not controlling for these potential confounding factors on TL (diet and sleep), another major limitation of the present study is that it lacks a control group. Although we report that the resistance training intervention had no observable change on LTL, we cannot be certain that a sedentary control group would have also displayed the same results over a 12-week period. For example, Dimauro et al. has previously reported that whilst TL remained unchanged in subjects performing a 3-month resistance training intervention, their telomeres were significantly longer than the sedentary control group after the study (Dimauro et al., 2016). However, this scenario is unlikely as it appears that sedentary individuals do not typically show any observable telomere attrition over such short intervention periods (Hagstrom, 2018; Tosevska et al., 2016; Werner et al., 2018). It is also possible that the small sample size may have affected the current results. Thus, longer intervention periods may be required to reveal the full potential benefits of resistance training on TL and the present study may serve to act as a pilot to a further investigation that utilises a control group, larger sample size and longer intervention period (>12 months). In support of a longer intervention period, there was a borderline significant increase in relative mRNA expression level of hTERT. Expression of hTERT mRNA has been reported to correlate with telomerase activity (Kirkpatrick et al., 2003), congruent with the notion that synthesis and expression of this subunit represents the rate-limiting determinant of telomerase activity (Counter et al., 1998). mRNA expression level of hTERT may have become significant had a 12-month resistance training intervention been employed, potentially reflecting increased telomerase activity, which could have had a positive influence on TL. Although we did not measure telomerase activity directly our findings support those of Werner et al., who recently demonstrated that telomerase activity was unchanged following a 6-month resistance training intervention (Werner et al., 2018). However, this conclusion should be interpreted with caution, as it assumes that hTERT mRNA expression level accurately represents telomerase activity.

In conclusion, the present study demonstrates that a 12-week low-resistance, high-repetition resistance training intervention does not cause any observable changes in LTL in healthy sedentary middle-aged subjects but does improve molecular and physical parameters associated with telomere dynamics. It appears that long-term, regular exercise (minimum of moderate score on IPAQ-SF >12-months) is necessary to preserve TL, a process which may be mediated by molecular pathways influenced by low-resistance, high-repetition resistance training. However, as with any study with such small sample size, our data should be interpreted with caution.

## Declarations

### Author contribution statement

M. Nickels: Conceived and designed the experiments; Performed the experiments; Analyzed and interpreted the data; Contributed reagents, materials, analysis tools or data; Wrote the paper.

E. Akam: Conceived and designed the experiments; Analyzed and interpreted the data.

D. Hunter: Performed the experiments.

M. Denniff: Performed the experiments; Contributed reagents, materials, analysis tools or data.

S. Mastana: Analyzed and interpreted the data.

V. Codd: Contributed reagents, materials, analysis tools or data.

### Funding statement

This work was supported by 10.13039/501100000857Loughborough University and Les Mills International, New Zealand.

### Competing interest statement

The authors declare no conflict of interest.

### Additional information

No additional information is available for this paper.
